# Heme Oxygenase-1 Inhibitors Induce Cell Cycle Arrest and Suppress Tumor Growth in Thyroid Cancer Cells

**DOI:** 10.3390/ijms19092502

**Published:** 2018-08-24

**Authors:** Po-Sheng Yang, Yi-Chiung Hsu, Jie-Jen Lee, Ming-Jen Chen, Shih-Yuan Huang, Shih-Ping Cheng

**Affiliations:** 1 Department of Surgery, MacKay Memorial Hospital and Mackay Medical College, Taipei 10449, Taiwan; yangpos@outlook.com (P.-S.Y.); leejj1957@pchome.com.tw (J.-J.L.); mjc@livemail.tw (M.-J.C.); shihyuanh@outlook.com (S.-Y.H.); 2 Department of Biomedical Sciences and Engineering, National Central University, Taoyuan City 32001, Taiwan; hsuyc@gmx.com; 3 Department of Pharmacology, School of Medicine, College of Medicine, Taipei Medical University, Taipei 11031, Taiwan

**Keywords:** heme oxygenase, metabolism, reactive oxygen species, thyroid cancer

## Abstract

Heme oxygenase-1 (HO-1) is induced by a variety of stimuli and plays a multifaceted role in cellular protection. We have shown that HO-1 is overexpressed in thyroid cancer and is associated with tumor aggressiveness. Therefore, we set out to assess the effects of HO-1 inhibitors on the biology of thyroid cancer cells. Two different classes of HO-1 inhibitors were used, including a metalloporphyrin, zinc protoporphyrin-IX (ZnPP), and an azole antifungal agent, ketoconazole. The viability and colony formation of thyroid cancer cells decreased in a concentration- and time-dependent fashion following treatment with HO-1 inhibitors. Cancer cells exhibited a higher sensitivity to HO-1 inhibitors than non-malignant cells. HO-1 inhibitors induced a G0/G1 arrest accompanied by decreased cyclin D1 and *CDK4* expressions and an increase in levels of p21 and p27. HO-1 inhibitors significantly increased intracellular ROS levels and suppressed cell migration and invasion. Oxygen consumption rate and mitochondrial mass were increased with ZnPP treatment. Mice treated with ZnPP had a reduced xenograft growth and diminished cyclin D1 and Ki-67 staining in tumor sections. Taken together, HO-1 inhibitors might have therapeutic potential for inducing cell cycle arrest and promoting growth suppression of thyroid cancer cells in vitro and in vivo.

## 1. Introduction

The heme oxygenase pathway is an important sensor of cellular stress and regulator of homeostasis [1]. Heme oxygenases are evolutionarily highly conserved enzymes that catalyze the degradation of heme to carbon monoxide, ferrous iron, and the bile pigment biliverdin, which in turn is reduced to bilirubin. In mammalian cells, there are two different isoforms of heme oxygenases: heme oxygenase-1 (HO-1) and HO-2. The expression of HO-1 is induced in response to a variety of endogenous and exogenous stimuli, whereas HO-2 is constitutively expressed.

HO-1 plays a pivotal and multifaceted role in cellular protection, which is likely attributable to its antioxidant, anti-inflammatory, and antiapoptotic properties. Nonetheless, the augmented expression of HO-1 in tumor tissues may have detrimental effects as it provides the selective advantage for tumor cells to overcome the increased oxidative stress during tumorigenesis and during treatment [2]. Recent experimental evidence suggests that the inhibition of expression or activity of HO-1 may contribute to an increased efficacy of chemotherapy and improve the outcome in animal models [3]. Nonetheless, the therapeutic benefits of HO-1 inhibition have not been tested in clinical trials.

The thyroid gland is characterized by an environment where oxidative stress is continuously produced during the steps of iodide metabolism and thyroid hormone synthesis. The oxidative stress may be linked to tumorigenesis and progression of thyroid cancer [4]. Strikingly, an alteration in the oxygen and reactive oxygen species (ROS) metabolic process has been identified during the progression of papillary thyroid cancer [5]. We recently demonstrated that expression of HO-1 in thyroid cancer was associated with an advanced tumor stage [6]. Therefore, it would be worthwhile exploring the therapeutic potential of HO-1 inhibitors in the management of thyroid cancer.

Some metalloporphyrins such as zinc protoporphyrin-IX (ZnPP) and tin protoporphyrin-IX can act as competitive inhibitors of heme oxygenases owing to their structural similarity to the heme substrate [7]. These inhibitors are extensively used in experimental studies including various murine models and were clinically used in patients with hyperbilirubinemia and hereditary porphyria [8,9]. Metalloporphyrins represent the first generation of HO-1 inhibitors, generally characterized by non-selectivity towards HO-1 and HO-2 isoforms. A previous report indicated that Zn compounds were least inhibitory toward HO-2 [10]. However, none of the metalloporphyrins were selective for HO-1. During the search for selective HO-1 inhibitors, researchers have synthesized a number of imidazole-dioxolane compounds (including compounds with inhibitory properties against HO that share structural features with azole-containing antifungal agents) which represent the second-generation inhibitors [11,12].

In the present study, we aimed to investigate the effects of these two different classes of pharmacological HO-1 inhibitors on thyroid cancer cells. Furthermore, we evaluated the efficacy and safety of ZnPP treatment using an in vivo model with xenografted nude mice.

## 2. Results

### 2.1. Suppression of Cell Viability and Clonogenic Ability by HO-1 Inhibitors

**Figure 1:**
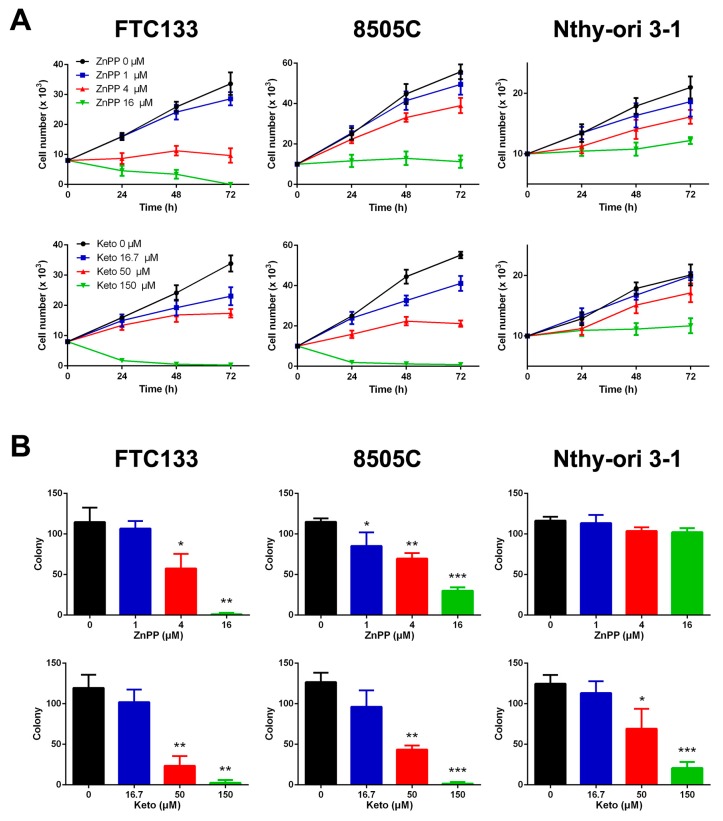
Decreased cell viability (**A**) and clonogenic ability (**B**) following treatment with heme oxygenase-1 inhibitors, zinc protoporphyrin-IX (ZnPP) and ketoconazole (Keto), in thyroid cancer cell lines (FTC-133 and 8505C) and a normal thyroid cell line (Nthy-ori 3-1). * *p* < 0.05 versus control, ** *p* < 0.01, *** *p* < 0.001.

The effects of two HO-1 inhibitors on cell viability was assessed in two thyroid cancer cell lines and a non-malignant human follicular thyroid line. These cell lines were validated to have moderate to high expression of HO-1 (Supplementary Materials, Figure S1). Treatment with ZnPP upregulated the HO-1 expression in both FTC-133 and 8505C cells, whereas treatment with ketoconazole at high doses slightly increased the HO-1 expression in 8505C cells only (Supplementary Materials, Figure S2). As shown in Figure 1A, the cell growth was significantly inhibited by ZnPP or ketoconazole in a dose-dependent manner in FTC-133 and 8505C thyroid cancer cells. The concentrations of ZnPP that caused 50% inhibition (IC_50_) at 72 h were 2.6 ± 0.3 and 6.9 ± 0.9 μM for FTC-133 and 8505C cells, respectively. These were significantly lower than the predicted IC_50_ of 17.9 ± 1.0 μM for Nthy-ori 3-1 cells (*p* < 0.0001 and *p* = 0.0002). Consistently, the IC_50_ values of ketoconazole for FTC-133 and 8505C cells were significantly lower than that of Nthy-ori 3-1 cells (44.7 ± 4.4 and 36.6 ± 1.3 μM versus 736.0 ± 257.1 μM; both *p* = 0.03).

A similar trend was observed using the colony formation assay which determines the ability of a single cell to grow into a colony. The number of colonies decreased with increasing doses of ZnPP or ketoconazole in FTC-133 and 8505C cells (Figure 1B). The non-malignant Nthy-ori 3-1 cells were sensitive to exposure to ketoconazole but not to ZnPP. The IC_50_ values of ZnPP for FTC-133 and 8505C cells were 5.4 ± 0.7 and 6.1 ± 0.9 μM, respectively. Nthy-ori 3-1 cells had a significantly higher IC_50_ of ketoconazole (62.1 ± 5.8 μM) than FTC-133 and 8505C cells (35.4 ± 7.1 and 37.3 ± 6.1 μM; both *p* = 0.03). Taken together, thyroid cancer cells appear to display a selective sensitivity to HO-1 inhibitors.

### 2.2. Cell Cycle Arrest Induced by HO-1 Inhibitors

**Figure 2:**
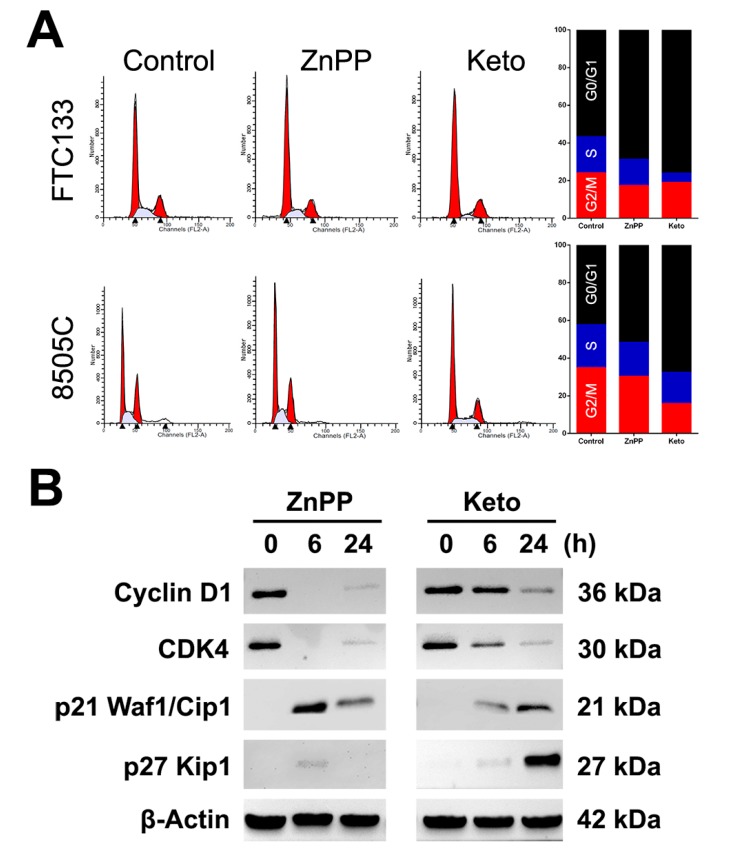
Effects of heme oxygenase-1 inhibitors, zinc protoporphyrin-IX (ZnPP) and ketoconazole (Keto), on cell cycle progression (**A**) and the expression of cell cycle regulators (**B**) in thyroid cancer cells.

The distribution of cell cycle phases was analyzed by flow cytometry in thyroid cancer cells treated with vehicle control, ZnPP (4 μM), or ketoconazole (50 μM). In FTC-133 cells, the percentage of G0/G1 phase cells increased from 56.7 ± 0.4% to 68.8 ± 2.3% and 76.1 ± 1.9% with the treatment of ZnPP and ketoconazole, respectively (*p* = 0.006 and 0.0005, Figure 2A). In 8505C cells, following the treatment with ZnPP or ketoconazole, the percentage of G0/G1 phase cells increased from 42.4 ± 1.8% to 51.7 ± 1.5% and 67.6 ± 0.4%, respectively (*p* = 0.02 and 0.0002). The number of sub-G0 cells and polyploid cells remained minimal. This suggests that HO-1 inhibitors induce a G0/G1 cell cycle arrest but do not trigger apoptosis or mitotic catastrophe in thyroid cancer cells.

The expression of cell cycle regulators was further evaluated following treatment with ZnPP or ketoconazole in FTC-133 cells. After treatment with HO-1 inhibitors, the expression of cyclin D1 and *CDK4* decreased over time (Figure 2B). Notably, the alteration in cell cycle regulators occurred at the earlier time point following treatment with ZnPP. On the other hand, the levels of cyclin-CDK inhibitors p21 Waf1/Cip1 and p27 Kip1 were increased. These observations are consistent with the G0/G1 arrest in the flow cytometric analysis.

### 2.3. ROS Induction by HO-1 Inhibitors

**Figure 3:**
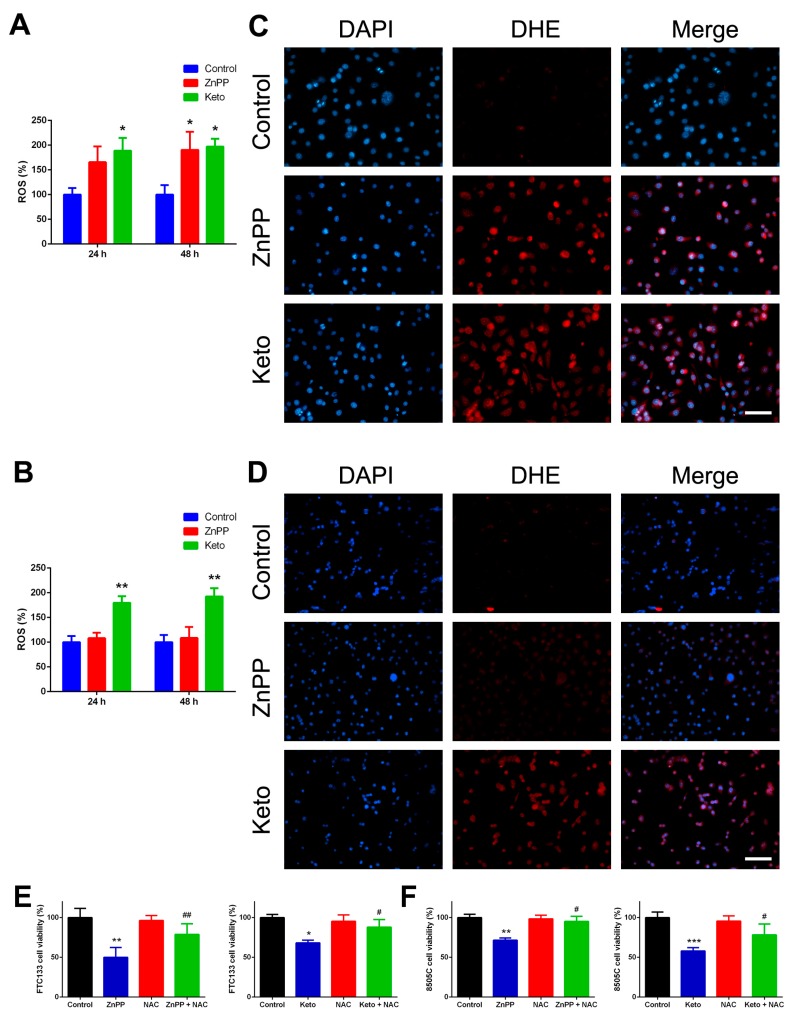
Reactive oxygen species (ROS) induction by heme oxygenase-1 inhibitors in thyroid cancer cells and effects of ROS scavenger. Following treatment with zinc protoporphyrin-IX (ZnPP), ketoconazole (Keto), or vehicle control, intracellular ROS was quantified by the Cellular Reactive Oxygen Species Detection Assay Kit (**A**,**B**) or by dihydroethidium (DHE) staining (**C**,**D**). * *p* < 0.05 versus control, ** *p* < 0.01. Scale bar, 100 μm. Cell viability was determined by the CyQUANT Cell Proliferation Assay following treatment with ZnPP, Keto, *N*-acetyl-l-cysteine (NAC), or cotreatment with heme oxygenase-1 inhibitors and NAC for 48 h. * *p* < 0.05 versus control, ** *p* < 0.01, *** *p* < 0.001. # *p* < 0.05 versus ZnPP or Keto only, ## *p* < 0.01.

HO-1 plays an important role in ROS scavenging, and HO-1 downregulation leads to the increase of ROS and DNA damage-induced checkpoint activation [13]. We analyzed the intracellular ROS induction by treating thyroid cancer cells with HO-1 inhibitors from 24 to 48 h. As shown in Figure 3A,B, ketoconazole significantly increased the ROS levels in both cell lines, while ZnPP treatment effectively increased ROS levels only in FTC-133 cells. The findings partially correspond with our cell viability data that indicated 8505C cells were less sensitive to the ZnPP treatment. The observations were confirmed with dihydroethidium (DHE) staining. Following treatment with vehicle control, ZnPP (4 μM), or ketoconazole (50 μM) for 24 h, strong DHE staining was observed in thyroid cancer cells incubated with HO-1 inhibitors (Figure 3C,D). These results indicate that HO-1 inhibitors induce an elevation of intracellular ROS levels.

We also examined the effects of a ROS scavenger, *N*-acetyl-l-cysteine (NAC), on cell viability. FTC-133 and 8505C thyroid cancer cells were treated with ZnPP, ketoconazole, or cotreatment with HO-1 inhibitors and NAC (10 mM) for 48 h. As shown in Figure 3E,F, the repressive effect of HO-1 inhibitors on cell growth was significantly rescued by NAC. The findings confirmed that the growth inhibition induced by ZnPP or ketoconazole is, at least partly, induced by increased intracellular ROS levels.

### 2.4. Suppression of Cell Migration and Invasion by HO-1 Inhibitors

**Figure 4:**
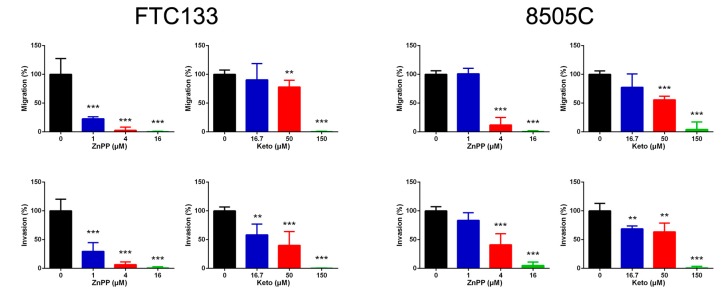
Cellular migration and invasion suppressed by heme oxygenase-1 inhibitors, zinc protoporphyrin-IX (ZnPP) and ketoconazole (Keto), in thyroid cancer cells. ** *p* < 0.01 versus control, *** *p* < 0.001.

We further investigated the effect of HO-1 inhibitors on other aspects of tumor biology. Using Transwell assays in which the number of cells that migrated across 8 μm diameter pores over 24 h was counted, we found that both ZnPP and ketoconazole steadily inhibited cellular migration and invasion in both thyroid cancer cell lines (Figure 4). These results suggest that HO-1 inhibitors not only attenuate cell viability but also impair in vitro cell motility.

### 2.5. Increased Mitochondrial Mass and Oxygen Consumption by HO-1 Inhibitors

**Figure 5:**
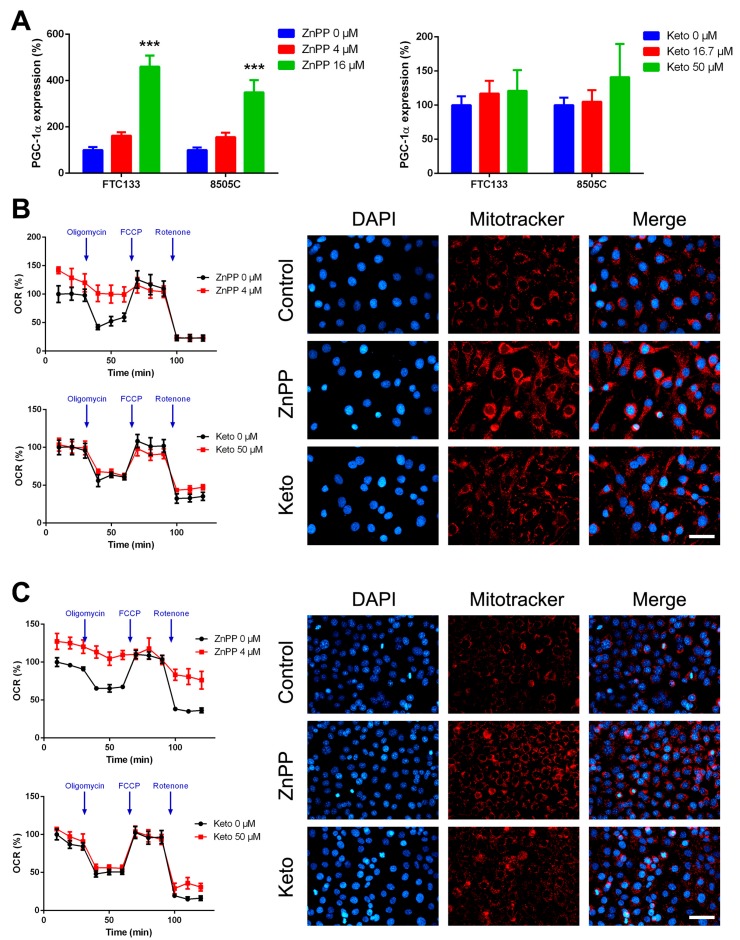
Metabolic alterations induced by heme oxygenase-1 inhibitors in thyroid cancer cells. The expression of peroxisome proliferator-activated receptor gamma coactivator 1-alpha (PGC-1α) was determined following treatment with zinc protoporphyrin-IX (ZnPP), ketoconazole (Keto), or vehicle control (**A**). Corresponding changes in oxygen consumption rates (OCR) and MitoTracker staining were evaluated in FTC-133 (**B**) and 8505C (**C**) cells. *** *p* < 0.001 versus control. Scale bar, 50 μm.

Previous studies demonstrated a possible association between HO-1 and peroxisome proliferator-activated receptor gamma coactivator 1-alpha (PGC-1α) expression [14,15]. The main function of PGC-1α is to control energy metabolism by facilitating oxidative metabolism, and particularly, mitochondrial oxidative phosphorylation. In addition to participating in energy and metabolic homeostasis, PGC-1α overexpression may reduce cell motility [16]. Following ZnPP treatment, the expression of PGC-1α was significantly upregulated in both thyroid cancer cell lines (Figure 5A). Nonetheless, a modest increase in PGC-1α expression after ketoconazole treatment did not attain significance.

Respiratory capacity was functionally validated. ZnPP treatment significantly increased basal but not maximal oxygen consumption rate (OCR) in FTC-133 and 8505C cells (Figure 5B,C). Furthermore, the response to an ATP synthase inhibitor, oligomycin, was blunted following treatment with ZnPP. In FTC-133 cells, the measured ECAR decreased from 18.3 ± 1.3 to 12.1 ± 0.8 mpH/min (*p* = 0.0007). In 8505C cells, the extracellular acidification rate (ECAR) decreased from 15.0 ± 0.7 to 12.6 ± 0.2 mpH/min (*p* = 0.006). Consistently, ZnPP-treated cells were stained intensely for MitoTracker, a marker of mitochondrial mass. These data indicated that HO-1 inhibitors lead to metabolic alterations toward a more oxidative phenotype in thyroid cancer cells.

### 2.6. Delayed Xenograft Tumor Growth with in Vivo ZnPP Treatment

**Figure 6:**
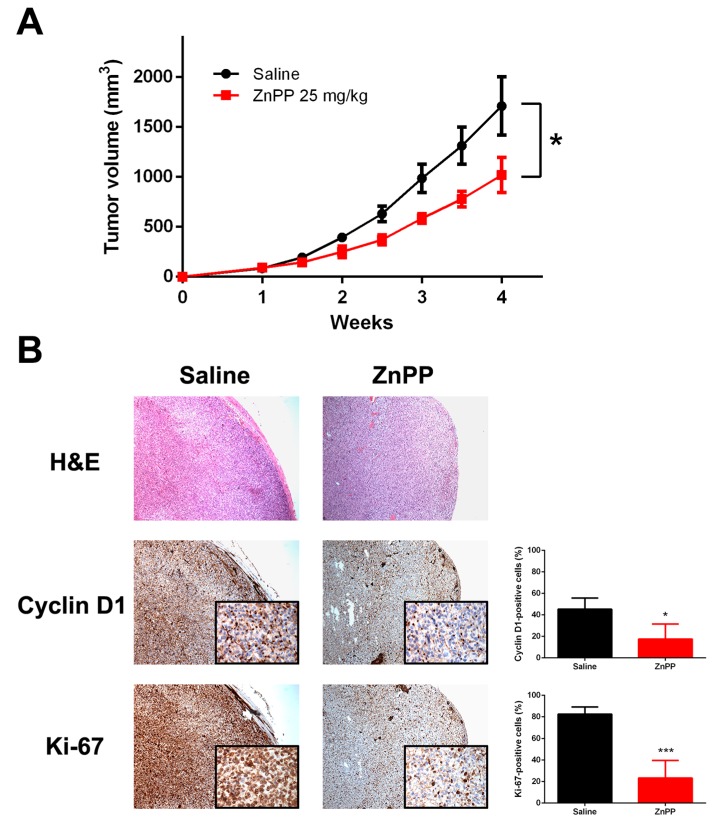
Xenograft tumor growth suppressed by the administration of a heme oxygenase-1 inhibitor. Nude mice were allocated to receive i.p. zinc protoporphyrin-IX (ZnPP) or saline control (*n* = 6 per group) following subcutaneous implantation of FTC-133 thyroid cancer cells, and the xenograft tumor volume was measured periodically (**A**). * *p* < 0.05 versus control. Representative microphotographs of tumor sections stained for H&E, cyclin D1, and Ki-67 are shown (**B**). Stained cells were manually counted from four separate areas on the slide and averaged per group. Original magnification ×40; inset, ×400. * *p* < 0.05 versus control, *** *p* < 0.001.

Mice were randomized into two groups: a control treated with saline and a treatment group, which was administered with i.p. 25 mg/kg ZnPP. As shown in Figure 6A, the FTC-133 xenograft tumor volume was significantly smaller in the treatment group by week 4. Throughout the treatment period, neither weight loss nor lethality was observed (Supplementary Materials, Figure S3). There was no tumor necrosis demonstrated by pathologic assessment of hematoxylin and eosin (H&E) stained sections of xenograft tumors. Interestingly, in agreement with in vitro observations, tumor sections in the treatment group showed a significant reduction in the cyclin D1 and Ki-67 staining (Figure 6B). Taken together, the present study showed that HO-1 inhibitors were effective in reducing tumor cell growth in cell-based studies and xenograft mouse models.

## 3. Discussion

Although differentiated thyroid cancer generally has an indolent course and excellent prognosis, some patients may experience locoregional recurrences and/or distant metastases which have negative impacts on survival [17]. The accumulation of mutations or epigenetic modifications during disease progression leads to genomic instability and increased ROS production. An upregulation of cell cycle-regulating and DNA repair genes may herald a higher recurrence risk in patients with papillary thyroid cancer [18]. The transcription factor Nrf2 is a key regulator of cellular antioxidant responses. In response to oxidative stress, nuclear Nrf2 activates antioxidant-responsive elements and stress-responsive target genes, including HO-1 [19]. It has been found that the Nrf2 expression and the oxidized lipid 4-hydroxy-2-nonenal (4-HNE) were more abundant in papillary thyroid cancer, and Nrf2 knockdown decreased the viability of thyroid cancer cells [20].

In this context, it is interesting to note that HO-1 was overexpressed in thyroid cancer and was associated with tumor aggressiveness [6]. Our previous findings were verified by a recent report showing that a 10-gene signature, including significantly overexpressed HO-1, may accurately classify indeterminate thyroid nodules [21]. While there has been a wealth of research on the prognostic roles of these antioxidant genes, little is known about the effectiveness of targeting ROS scavengers. A previous study suggests that HO-1 stimulated by hemin or cadmium may protect thyroid cancer cells from tumor necrosis factor-α and cycloheximide-induced apoptosis [22]. In this study, we found that HO-1 inhibitors induced cell cycle arrest via inhibition of cyclin-dependent kinases and induction of the CDK inhibitors p21 Waf1/Cip1 and p27 Kip1. Consistent with our findings, the effects of ROS induction and the decrease in cyclin D1 expression by ZnPP treatment have been described in other tumor cell types [23]. Intriguingly, the effects on cell viability were more prominent in thyroid cancer cells than non-malignant Nthy-ori 3-1 cells. Nonetheless, the inhibitory effects on xenograft tumor growth were modest without tumor regression, suggesting that it may be inappropriate to use HO-1 inhibitors alone as a sole agent for the management of thyroid cancer in the clinical setting. The effects of combining other treatment modalities, including radioactive iodine and tyrosine kinase inhibitors, remain to be elucidated.

A novel finding in our study is that HO-1 inhibitors are associated with metabolic reprogramming in thyroid cancer cells. The Warburg effect of aerobic glycolysis by converting pyruvate to lactate may reduce the intracellular oxidative stress in rapidly proliferating cancer cells [24]. Liver-specific HO-1 knockout hepatocytes exhibited increased respiratory capacity, supporting that HO-1 suppresses oxidative phosphorylation [25]. It has been shown that the increases in intracellular ROS levels can inactivate the glycolytic enzyme pyruvate kinase M2 [26]. In the present study, we found that HO-1 inhibitors increased the intracellular ROS levels and upregulated the expression of PGC-1α, resulting in a switch from the glycolytic metabolic profile to a more oxidative phenotype. Fluorine-18-fluorodeoxyglucose (^18^F-FDG)-avid thyroid cancers represent a dedifferentiated, aggressive, and metabolically active subgroup and thereby indicate poorer patient outcomes [27]. An enhanced glycolysis profiling in elder patients may partially explain the phenomenon of age-dependent prognosis in thyroid cancer [28]. In this regard, the metabolic reprogramming effects of HO-1 inhibitors surely deserves attention. However, the effects appeared subtle with ketoconazole treatment, and it is unclear whether this finding is unique to thyroid cancer.

We also observed that HO-1 inhibitors attenuated migration and invasion of thyroid cancer cells. This is in agreement with a previous report showing that HO-1 knockdown inhibited transwell cell migration in cholangiocarcinoma cells [8]. Although it was reported that the HO-1-mediated migration/invasion may be ascribed to intramembrane proteolysis and nuclear translocation, which are independent of its enzymatic activity [29], our results suggest that the HO-1 activity or the products of heme degradation can be pharmacologically targeted to suppress cell motility in thyroid cancer. Additionally, PGC-1α overexpression may upregulate E-cadherin expression and lower the cell migratory ability [16]. Nonetheless, ketoconazole had subtle effects on PGC-1α expression but retained the inhibitory activity for cell motility in our study. More studies are needed to unravel the underlying mechanisms.

Our results, however, should be interpreted with caution. Although the observations were carried out on two different classes of HO-1 inhibitors, we cannot exclude the possibility of off-target effects from these HO-1 inhibitors because some heterogeneous responses were observed among two inhibitors. Moreover, rigorous mechanistic studies are needed to ascertain which factors are responsible for the major effects: reduced HO-1 activity and reaction products, changes in oxidative stress, or metabolic alterations. Additional work is required to examine the effects of these inhibitors on in vivo tumor biology (e.g., metastatic spread) in more detail and whether there are synergic effects of combining HO-1 inhibitors with other types of therapy. Nonetheless, our study provides evidence that HO-1 overexpression in thyroid cancer has the potential not only to be prognostic but also to present a therapeutic target. Cancer cells possess an increased antioxidant capacity to counteract elevated ROS levels. Our results corroborate the view that suppression of oxidative stress-coping machinery of cancer cells represents a promising strategy in anticancer therapy [30].

Collectively, we identified that HO-1 inhibitors might have therapeutic potential for inducing cell cycle arrest and promoting growth suppression of thyroid cancer cells in vitro and in vivo. Nonetheless, a number of challenges will have to be overcome to enable the translation of experimental findings to clinical medicine.

## 4. Materials and Methods

### 4.1. Cell Lines and Reagents

Human thyroid cancer cell lines (FTC-133 and 8505C) and an SV40 large T-immortalized human differentiated thyrocyte cell line (Nthy-ori 3-1) were obtained from the European Collection of Authenticated Cell Cultures, Salisbury, UK. Both FTC-133 and 8505C cell lines have been validated as authentic cell lines of thyroid origin [31]. FTC-133 cells were cultured in DMEM: Ham’s F12 (1:1), supplemented with 10% fetal bovine serum (FBS) and glutamine (2 mM). 8505C and Nthy-ori 3-1 cells were cultured in RPMI-1640 medium supplemented with 10% FBS and glutamine. In our laboratory, cell lines are regularly authenticated by DNA-short tandem repeat genotyping at Bioresource Collection and Research Center, Hsinchu, Taiwan, and tested for being mycoplasma-free. ZnPP, ketoconazole, and NAC were purchased from Sigma-Aldrich (Merck KGaA, Darmstadt, Germany).

### 4.2. Cell Growth and Colony Formation

Cell growth was determined by CyQUANT Cell Proliferation Assay (Thermo Fisher Scientific, Waltham, MA, USA) as specified by the manufacturer’s instructions. Briefly, cells were seeded on 96-well plates and treated with the indicated concentrations of ZnPP, ketoconazole, or vehicle control (dimethyl sulfoxide) for 24 to 72 h. At indicated time points, the medium was removed, and plates were stored at −80 °C. RNA fluorescence was eliminated by pretreating cell lysates with DNase-free RNase. Following adding CyQUANT GR dye/cell-lysis buffer, cell growth was determined by measuring the fluorescence intensity at 480 nm excitation and 520 nm emission.

Clonogenic ability was assessed as previously described [32]. A total of 500 FTC-133, 8505C, or Nthy-ori 3-1 cells were seeded into six-well plates in growth medium with the indicated concentrations of ZnPP, ketoconazole, or vehicle control. The medium was replaced every 3 days. After 7–12 days, colonies were stained with 3% crystal violet and counted. 

### 4.3. Reverse-Transcription Polymerase Chain Reaction

Total RNA was prepared with TRIzol reagent (Thermo Fisher Scientific), and cDNA was synthesized and further PCR-amplified by specific primers. The amplified products were visualized on agarose gels with ethidium bromide after electrophoresis or analyzed on an Applied Biosystems 7500 Fast Real-Time PCR System [33]. The following primers were used in this study: HMOX1_F, CTCAAACCTCCAAAAGCC; HMOX1_R, TCAAAAACCACCCCAACCC; PPARGC1A_F, AACAGCAGCAGAGACAAATGCACC; PPARGC1A_R, TGCAGTTCCAGAGAGTTCCACACT.

### 4.4. Cell Cycle Analysis

The effects of HO-1 inhibitors on cell cycle progression were evaluated by flow cytometry as described previously [34]. Cells were synchronized by serum deprivation for 24 h and then treated with the indicated concentrations of ZnPP or ketoconazole for 6, 24 and 48 h. Following treatment, cells were harvested, washed, and fixed in 70% cold ethanol at 4 °C overnight. The fixed cells were stained with propidium iodide solution using the BD Cycletest Plus DNA Kit (BD Biosciences, San Jose, CA, USA) and analyzed for DNA content on a FACSCalibur flow cytometer (BD Biosciences) equipped with CellQuest Pro software (version 5.1; BD Biosciences). The percentage of the cells in different phases of the cell cycle and the percentage of apoptotic cells were determined using the ModFit LT software (Verity Software House, Topsham, ME, USA).

### 4.5. Western Blotting

Total cellular proteins were extracted, quantified, and subjected to gel electrophoresis according to standard procedures as we described previously [35]. The antibodies used in this study included anti-HO-1 (ab52947; Abcam, Cambridge, UK), anti-Cyclin D1 (#2978), anti-CDK4 (#12790), anti-p21 Waf1/Cip1 (#2947), anti-p27 Kip1 (#3686), and anti-actin (A5441; Sigma-Aldrich). Antibodies were obtained from Cell Signaling Technology, Danvers, MA, USA, unless specified otherwise. Immunoreactive bands were detected by enhanced chemiluminescence (Amersham ECL Detection System; GE Healthcare, Piscataway, NJ, USA).

### 4.6. ROS Detection Assays

The measurement of intracellular ROS was determined by the Cellular Reactive Oxygen Species Detection Assay Kit (Abcam) as per the manufacturer’s instructions. To detect ROS levels, the cell-permeant, non-fluorescent 2′,7′-dichlorofluorescein diacetate (DCFDA) is oxidized by hydroxyl, peroxyl or other ROS to fluorescent DCF. Cells were stained with DCFDA or assay buffer as a negative control, and then treated with ZnPP, ketoconazole, or vehicle control in the dark for the indicated times. Fluorescence was detected with excitation and emission wavelengths at 485 and 535 nm, respectively. Fluorescence DCF values were normalized to the corresponding cell viability data.

The superoxide generation in the mitochondria was further visualized by DHE (Sigma-Aldrich) staining. DHE freely enters cells and is oxidized by superoxide to ethidium bromide which emits red fluorescence. Cells were treated with ZnPP, ketoconazole, or vehicle control for 24 h, and then fixed and stained with DHE for 30 min. Nuclear counterstaining was performed using 4′,6-diamidino-2-phenylindole (DAPI; Sigma-Aldrich). Samples were examined under a fluorescence microscope.

### 4.7. Transwell Migration and Invasion Assays

Cellular migration and invasion assays were performed using the Boyden chamber system with a pore size of 8 μm (Corning Life Sciences, Corning, NY, USA). For invasion assays, inserts coated with Matrigel matrix were used [36]. Cells were seeded onto inserts in 1%-FBS medium containing ZnPP, ketoconazole, or vehicle control. The lower wells were filled with the complete medium with the same concentration of HO-1 inhibitors. The plates were incubated for 24 h. Cells on the lower surface of the membrane were fixed, stained using the Diff-Quik staining kit (Sysmex, Kobe, Japan), and counted.

### 4.8. Oxygen Consumption

OCR was determined using the XF24 Extracellular Flux Analyzer (Seahorse Bioscience; Agilent Technologies, North Billerica, MA, USA). Cells were seeded in Seahorse XF24 microplates and cultured overnight to allow full attachment. The following day, the cell culture medium was replaced with fresh medium containing the indicated concentrations of ZnPP or ketoconazole for 24 h. On the day of the assay, all media and injection reagents were adjusted to pH 7.4. Three baseline measurements of the OCR and ECAR were taken and subsequently in the presence of the ATP synthase inhibitor oligomycin (1 μM), protonophore uncoupler carbonyl cyanide 4-(trifluoromethoxy) phenylhydrazone (FCCP, 1 μM), and mitochondrial respiratory chain complex I inhibitor rotenone (2 μM).

### 4.9. MitoTracker Red Staining

Following treatment with ZnPP, ketoconazole, or vehicle control for 48 h, cells were fixed and stained with MitoTracker Red CMXRos (Thermo Fisher Scientific) for 30 min. DAPI counterstaining was performed to visualize the nuclei. Fluorescent images were captured by a fluorescence microscope.

### 4.10. Xenograft Implantation

All animal procedures were approved by the Animal Care and Use Committee of the institution in compliance with the Guide for the Care and Use of Laboratory Animals. Six-week-old female BALB/c nude mice were purchased from the National Laboratory Animal Center, Taipei, Taiwan. FTC-133 cells (1 × 10^7^) were injected subcutaneously into the bilateral flank of mice [37]. One week after tumor inoculation, mice were allocated to the treatment group (ZnPP 25 mg/kg body weight i.p. once daily, 5 days per week) or the control group (treated with the same volume of saline control). Tumor volume was calculated as length × width^2^ × 0.5. Body weight was measured weekly. Mice were euthanized when humane endpoints were reached. Xenograft tumors were harvested, fixed, and paraffin embedded for H&E and immunohistochemical staining.

### 4.11. Immunohistochemistry

Formalin-fixed paraffin-embedded xenograft tumor sections were subjected to deparaffinization, rehydration, and antigen retrieval for immunohistochemical staining as described previously [38]. Primary antibodies used for immunohistochemical staining were rabbit monoclonal antibodies to cyclin D1 (RM-2113-R7; Thermo Fisher Scientific) and Ki-67 (RM-9106-R7; Thermo Fisher Scientific).

### 4.12. Statistical Analysis

The data are expressed as means ± standard errors of the mean. Statistical analysis was performed with the two-tailed Student *t* test or one-way analysis of variance with Holm-Sidak post hoc test for multiple comparisons. A *p* value of <0.05 was considered statistically significant.

## Supplementary Materials

The following are available online at http://www.mdpi.com/1422-0067/19/9/2502/s1.

## Abbreviations

HO-1	heme oxygenase-1
ROS	reactive oxygen species
ZnPP	zinc protoporphyrin-IX
IC_50_	The concentration causes 50% inhibition
DHE	dihydroethidium
NAC	*N*-Acetyl-l-cysteine
PGC-1α	peroxisome proliferator-activated receptor gamma coactivator 1-alpha
OCR	oxygen consumption rate
ECAR	extracellular acidification rate
FBS	fetal bovine serum
DCFDA	2′,7′-dichlorofluorescein diacetate
DAPI	4′,6-diamidino-2-phenylindole
H&E	hematoxylin and eosin
