# Supplementary material for: Heme Oxygenase-1 Inhibitors Induce Cell Cycle Arrest and Suppress Tumor Growth in Thyroid Cancer Cells
Source: Int J Mol Sci. 2018 Aug 24;19(9):2502. doi: 10.3390/ijms19092502 (PMC6163304; doi:10.3390/ijms19092502)
Supplement: Supplementary file 1 [file ijms-19-02502-s001.pdf]

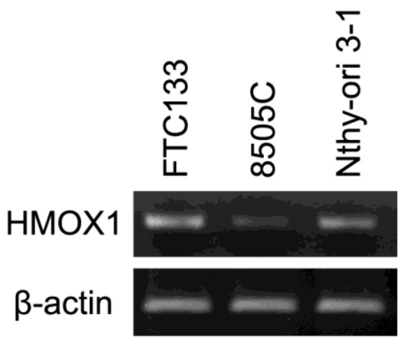

Figure S1. RNA expression of heme oxygenase-1 (HMOX1) in thyroid cancer cell lines (FTC-133 and 8505C) and a normal thyroid cell line (Nthy-ori 3-1).

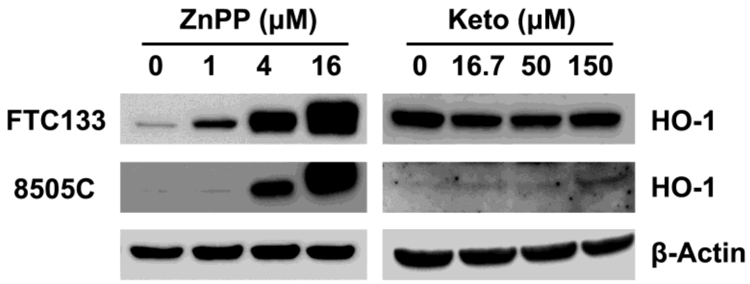

Figure S2. Expression of heme oxygenase-1 (HO-1) in thyroid cancer cell lines (FTC-133 and 8505C) following treatment with HO-1 inhibitors, zinc protoporphyrin-IX (ZnPP) and ketoconazole (Keto), for 24 h.

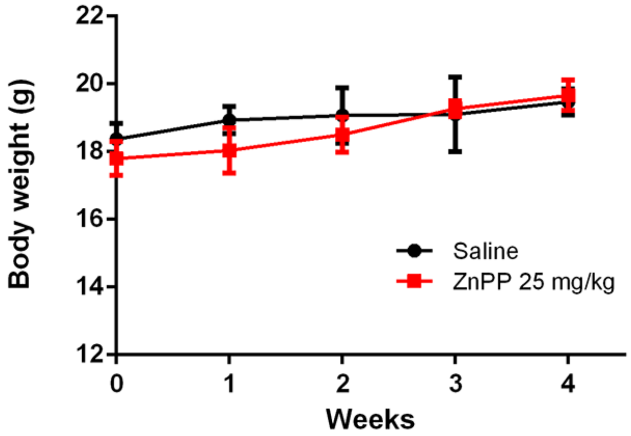

Figure S3. Changes in body weight following treatment with zinc protoporphyrin-IX (ZnPP) or saline control in nude mice with subcutaneous implantation of FTC-133 thyroid cancer cells.
